# The US Distribution of Physicians from Lower Income Countries

**DOI:** 10.1371/journal.pone.0033076

**Published:** 2012-03-21

**Authors:** E. Fuller Torrey, Barbara Boyle Torrey

**Affiliations:** 1 Stanley Medical Research Institute, Chevy Chase, Maryland, United States of America; 2 National Research Council, Bethesda, Maryland, United States of America; Aga Khan University, Pakistan

## Abstract

**Introduction:**

Since the 1960 s, the number of international medical graduates (IMGs) in the United States has increased significantly. Given concerns regarding the effects of this loss to their countries of origin, the authors undertook a study of IMGs from lower income countries currently practicing in the United States.

**Methods:**

The AMA Physician Masterfile was accessed to identify all 265,851 IMGs in active practice in the United States. These were divided by state of practice and country of origin. World Bank income classification was used to identify lower income countries.

**Results:**

128,729 IMGs were identified from 53 lower income countries, constituting 15 percent of the US active physician workforce. As a percentage of the workforce, West Virginia (29%), New Jersey (27%), and Michigan (26%) had the most IMGs from lower income countries, and Montana, Idaho, and Alaska (all less than 2%), the least. The countries with the greatest loss of physicians to the United States per 100,000 population were the Philippines, Syria, Jordan, and Haiti.

**Discussion:**

The reliance of US medicine on physicians from lower income countries is beneficial to the United States both clinically and economically. However, it results in a loss of the lower income country's investment in the IMG's education. We discuss possible mechanisms to compensate the lower income countries for the medical education costs of their physicians who immigrate to the US.

## Introduction

International medical graduates (IMGs) in the United States have become increasingly important over the past half century. In 1965 IMGs constituted approximately 10 percent of the physician workforce [1]; in 1981, this had increased to 21 percent, and today is 32 percent [2], [3]. According to a 2005 study, 12 percent of all IMGs practicing in the United States are US citizens who went abroad for training and then returned [4]. IMGs also constitute a significant part of the physician workforce in other developed countries such as Canada (23.1 percent), Australia (26.5 percent), and the United Kingdom (28.3 percent) [4].

Physicians migrate to other countries for many reasons, including better salaries, working conditions, living conditions, safety, opportunities for advancement, and participation in research. Such factors may be either “push” (conditions in the country of origin) or “pull” (conditions in the target country) factors. An example of the latter is immigration regulations, such as the liberalized visa requirements for highly skilled workers from China and India recently passed by the US House of Representatives [5].

In recent years, concerns have been expressed by organizations such as the US Institute of Medicine [6], the World Health Organization [7], and independent commissions [8] regarding the effects of physician losses on lower income countries. Countries in sub-Saharan Africa, for example, have 24 percent of the global burden of disease but only 3 percent of the global medical workforce [9]. We therefore undertook a study of the number and distribution by state of IMGs from lower income countries. The loss of medical training investment in lower income countries is compared to the American health-related foreign aid to that country. Finally, we discuss possible mechanisms to minimize health investment losses to the lower income countries that are contributing physicians to the United States.

## Methods

The AMA Physician Masterfile [10] was accessed in December 2010 to identify all 265,851 physicians currently practicing in the United States who received their training in medical schools in other countries. These included only physicians currently in practice and not those who are retired or otherwise not practicing. The database provided the medical schools and countries of training as well as the states in which the physicians are practicing. The World Bank income per capita classification was used to identify those countries defined as low income (gross national income per capita of $975 or less) and lower middle income ($976–$3,855) [11]. For analysis purposes, low income and lower middle income countries are jointly referred to as lower income countries.

To access implications of this physician migration from lower income countries to the United States, we sought information on the costs of training physicians in lower income countries [8]. We then compared that with published data on health-related foreign aid given by the United States to these same countries [12], [13].

## Results

IMGs currently practicing medicine in the United States come from 29 of the 42 low income countries, as defined by the World Bank. Thirty-nine of the 57 lower middle income countries have physicians trained in their countries practicing in the US. However, for 15 of the countries, the number of physicians was six or fewer, so these countries were omitted from the study. This left 7,946 physicians from 19 low income countries and 120,783 physicians from 34 lower middle income countries, or 128,729 physicians total. Thus, more than 48 percent of all IMGs practicing in the United States, and 15.4 percent of all active US physicians, are from lower income countries that have the least adequate health systems in the world.

The origin and distribution of the IMGs from lower income countries are both concentrated; 85 percent of them come from just 8 countries, and 67 percent of all IMGs are living in just 10 states (see Table S1). Forty-one percent of all IMGs from lower income countries come from India, and 22 percent of them are practicing in New York and California. The Philippines is the second largest provider of physicians from lower income countries (16 percent), and they are also practicing in disproportionate numbers in New York and California. Physicians trained in Pakistan, the third most important country of origin of IMGs in the United States (10 percent), practice disproportionately in Texas, New York, and Illinois.

The dependency on IMGs from lower income countries by state can be quantified by looking at their number relative to the total practicing physicians in each state. Twenty-nine percent of practicing physicians in West Virginia are from lower income countries. New Jersey, Minnesota, and Illinois have the next highest rates of dependency, at 27 percent, 26 percent, and 24 percent, respectively.

There is also a significant geographical clustering of IMGs from lower income countries in the United States by their country of origin (Table S1). Sixty-nine percent of all Haitian IMGs practice in New York and Florida; 47 percent of IMGs from Vietnam and 38 percent of those from Myanmar practice in California; and 34 percent of Ethiopian IMGs work in the greater Washington area (the District of Columbia, Maryland, and Virginia). Fifty-five percent of all lower income country IMGs in Hawaii were trained in the Philippines, and 56 percent of IMGs in Nebraska were trained in India.

Regarding the implications of having large numbers of IMGs from lower income countries practicing in the United States, relatively little comparable data is available on the costs of medical education in other countries. Such costs may vary widely within a country and between public and private medical schools [14]. The Commission on Education of Health Professionals for the 21^st^ Century recently estimated the average medical graduate costs to be $14,000 in China; $35,000 in India; $52,000 in sub-Saharan Africa; $113,000 in North Africa/Middle East; $132,000 in Latin America/Caribbean; and $151,000 in Eastern Europe, compared to $497,000 in North America [8]. Multiplying medical graduate costs for each country or region by the number of IMGs from each lower income country provides an approximate estimated loss of the medical education investment from that country (Table 1). This can then be compared with the total health-related foreign aid that was given by the US to these countries in fiscal year 2010. As shown in Table 1, the training costs for lower income countries' medical graduates working in the US exceeds the total health-related foreign aid being given by the US to these countries in 19 of the 39 countries. The most extreme examples are India and the Philippines. The comparison is, of course, imperfect, since it compares the training costs of IMGs who have arrived over many years with the health-related foreign aid for a single year, but it illustrates the magnitude of the loss for some countries. In addition to financial losses suffered by a lower income country when a physician leaves, there are other losses. These include the loss of a role model for young people, the loss of a mentor for physicians in training, the loss of employment for health service workers who would have been employed by the physician, and the loss of the physician's medical services.

**Table 1:** Training Costs of IMGs Compared to US Foreign Aid for Health by Country.

	IMGs in US	Cost of training (in thousands) [8]	Total cost of training (in millions)	AID(in millions)[12]	State Dept (in millions) [13]	Total(in millions)	Total training costs less foreign aid (in millions)
Armenia	315	$85	$26.8	$0.4	0	$0.4	$26.4
Cameroon	63	$52	$3.3	$1.5	$1.3	$2.8	$0.5
China	5,584	$14	$78.2	$4.0	$3.0	$7.0	$71.2
El Salvador	373	$132	$49.2	$5.5	0	$5.5	$43.7
Georgia	208	$85	$17.7	0	$0.9	$0.9	$16.8
Guatemala	558	$132	$73.7	$14.6	0	$14.6	$59.1
Honduras	166	$132	$21.9	$11.0	$1.0	$12.0	$9.9
India	5,2874	$35	$1,850.6	$78.2	$9.0	$87.2	$1,763.4
Kyrgyz Republic	30	$74	$2.2	$1.2	$0.5	$1.7	$0.5
Nicaragua	330	$132	$43.6	$5.9	$0.9	$6.8	$36.8
Pakistan	12,433	$35	$435.2	$29.7	0	$29.7	$405.5
Paraguay	263	$132	$34.7	$2.1	0	$2.1	$32.6
Philippines	20,625	$85	$1,753.1	$33.2	0	$33.2	$1,719.9
Sudan	353	$113	$39.9	$30.0	$7.0	$37.0	$2.9
Tajikistan	55	$74	$4.1	$1.5	$0.5	$2.0	$2.1
Thailand	1,688	$85	$143.5	$1.0	$0.5	$1.5	$142.0
Ukraine	1,560	$151	$235.6	$4.0	$14.7	$18.7	$216.9
Uzbekistan	124	$74	$9.2	$2.4	$0.6	$3.0	$6.2
Vietnam	1,164	$85	$98.9	0	$95.0	$95.0	$3.9

Note: This table includes only those lower income countries that received US bilateral foreign aid related to health and whose IMG training costs were larger than the aid. The US foreign aid totals are those enacted by Congress for fiscal year 2010.

## Discussion

The use of IMGs is beneficial to the United States for many reasons. IMGs are more likely than US medical graduates to be generalists, as opposed to specialists [2]; to practice in nonurban, primary care shortage areas [15]; and to treat more Medicaid patients [16]. IMGs practicing in the US may also benefit their country of origin by sending remittances home, thus partially offsetting the countries' economic losses [17]. A few lower income countries, such as the Philippines and Egypt, have at least implicitly encouraged the outmigration of physicians, nurses, engineers, and other professionals as a way to obtain hard currency remittances. For the Philippines in 2004, such remittances were estimated to represent 10 percent of the country's gross domestic product [18]. However, such remittances go to individual families and do nothing to compensate the health sector for the physician's training and loss of skills.

At the same time, it should be recognized that the loss of physicians by lower income countries makes it much more difficult for such countries to improve people's health. This appears to be especially true for countries in sub-Saharan Africa. WHO has estimated that 63 percent of the 57 countries that have the most severe health workforce crisis are in Africa [19]. One estimate suggested that the shortage of doctors in sub-Saharan Africa by 2015 will be 240,000, which makes the trends in their emigration particularly important [20]. In 2002 there were 5,334 African-trained IMGs in the US [21]; in 2010 the number had grown by 10 percent to 5,847. But the increase in African IMGs in the US has been much more from some countries, especially English-speaking countries, than others. The number of physicians trained in Kenya who immigrated to the United States increased 92 percent in those eight years. IMGs from Ghana increased 52 percent; from Nigeria, 58 percent; from Zimbabwe 63 percent; and from Ethiopia, over 100 percent.

While the percentage increases are large, the actual numbers of IMGs are relatively small compared to all the physicians in the US. However, when the number of African IMGs is compared to the number of physicians left in their country, the burden of emigration becomes clearer. Ghana has approximately 2,600 practicing physicians in the country compared with 532 in the US in (2004) and 259 more in Britain and Canada [22]. In Zambia the medical school in Lusaka trained over 600 medical graduates from 1977 to 2000; yet in 2000 only 50 of them were working in the Zambian public-sector health service [23]. As of 2006, there were almost as many Ethiopian physicians in the United States (542) as were practicing in the public sector in Ethiopia (638) [24].

A number of remedies have been proposed to deal with issues of IMGs in the United States. In the 1990 s, some organizations recommended that the US government restrict the entry and training of IMGs in the US [6]. Subsequently, however, US medicine became more, not less, dependent as IMG numbers continued to increased. Consequently, alternative solutions have been proposed in more recent years.

In 2001 the United Nations' Millennium Development Goals focused world attention on improving health in lower income countries and on strengthening their current health workforces [25]. The WHO's *2006 World Health Report* highlighted the emigration of IMGs and its effect on the health workforce on the countries with the largest health burdens [19]. The independent Commission on Education of Health Professionals for the 21^st^ Century made ten recommendations, including strengthening educational resources in poor countries, better use of information technology, and better networks [8]. In 2010 the World Health Assembly adopted the WHO Global Code of Practice on the International Recruitment of Health Personnel, which provides an ethical framework for recruiting countries and organizations to discourage emigration [26]. Several high-income countries have adopted similar codes. The problem is that the proposed solutions assume the altruism of potential IMGs to forego earning more money in wealthy countries. They also assume that the recruiting countries will act ethically and against their own self-interest.

Some people have suggested that there is an ethical obligation for the payment of restitution by high-income countries to lower income countries for the emigration of their physicians [27], [28]. This is theoretically appealing, but the issues of how to calculate restitutions given the wide variances in training costs make the proposal problematic. It could also create incentives for medical schools in lower income countries to increase their costs so that the restitution would be larger. In addition, it raises questions of whether such restitution could take into account the productivity of the IMG, which is likely to vary considerably.

Several recent proposals that directly target the African diaspora may be more practical. In 2009 a joint statement by the African Science Academies addressed the issues of all of Africa's scientists, including physicians [29]. They counseled acceptance of the fact that most African scientists living in higher income countries are unlikely to return. However, collaboration of international centers of excellence would be possible and a way of integrating the diaspora with the institutions back home. They also suggested programs that would attract emigrant scientists back to their homes for short periods of work with their peers who have stayed in the country. In 2010 the Sub-Saharan African Medical School Study reported that most African medical schools have some kind of international partnership with institutions in higher income countries and encouraged such arrangments [14]. These partnerships could help develop the capacity of medical schools in lower income countries, allow for opportunities for African physicians to further their training for short periods abroad, and provide research opportunities in lower income countries [30].

Independently, the US National Institutes of Health has partnered with other US agencies to develop the Medical Education Partnership Initiative [9]. Twenty-seven medical schools in thirteen African countries have already partnered with leading American and Canadian medical schools to provide technical expertise and to leverage resources. However, this represents only *14 percent* of the medical schools in Africa today and thus is just a beginning to what could become a more integrated system of IMGs and medical schools around the world.

The distribution of IMGs from lower income countries in the American states could be used to help build partnerships between their medical schools of origin and medical schools in the states. States that have a large number of IMGs from a particular country would have a comparative advantage in partnering with the medical schools in that country. It would provide a way for IMGs to return to their country to share their expertise as well as for American medical graduates to become more involved in international health problems. WHO could draw up a global matching formula based on the international distribution of IMGs by country. Lower income countries would set priorities among the most needy medical schools in their own countries. This would not be costless, but it need not be expensive. Logistics would be challenging but also an adventure aided by a digital world. Some partnerships would work better than others, but in most cases any help would be better than none.

The main limitation of the present study is that it undercounts physicians from lower income countries currently in the United States by including only those in active medical practice, not those who have retired. Nor does it include those who came to the United States but could not pass the requisite examinations and are now working in other jobs. In addition, it does not include IMGs from lower income countries in other countries and thus significantly underestimates the effects of the total physician losses on the lower income countries.

## Supporting Information

Table S1 **Distribution of International Medical Graduates by country of origin and US state of practice by absolute number and as a percentage of all state physicians, 2010.**
(DOC)
