# Supplementary material for: The US Distribution of Physicians from Lower Income Countries
Source: PLoS One. 2012 Mar 21;7(3):e33076. doi: 10.1371/journal.pone.0033076 (PMC3310056; doi:10.1371/journal.pone.0033076)
Supplement: Table S1 — Distribution of International Medical Graduates by country of origin and US state of practice by absolute number and as a percentage of all state physicians, 2010. (DOC) [file pone.0033076.s001.doc]

Table S1: Distribution of International Medical Graduates by country of origin and US state of practice by absolute number and as a percentage of all state physicians, 2010

| Country/State | Alabama | Alaska | Arizona | Arkansas | California | Colorado | Conn. | Delaware | District of Columbia | Florida | Georgia | Hawaii | Idaho | Iowa |
| --- | --- | --- | --- | --- | --- | --- | --- | --- | --- | --- | --- | --- | --- | --- |
| A. Lower Income |  |  |  |  |  |  |  |  |  |  |  |  |  |  |
| Afghanistan | 1 | 0 | 4 | 0 | 61 | 2 | 3 | 1 | 1 | 7 | 3 | 0 | 0 | 2 |
| Bangladesh | 25 | 0 | 23 | 5 | 65 | 3 | 16 | 4 | 8 | 71 | 45 | 1 | 0 | 6 |
| Ethiopia | 1 | 0 | 12 | 0 | 29 | 2 | 2 | 0 | 28 | 14 | 19 | 0 | 0 | 1 |
| Ghana | 6 | 0 | 16 | 5 | 20 | 0 | 37 | 5 | 12 | 20 | 29 | 0 | 1 | 5 |
| Haiti | 5 | 0 | 2 | 3 | 4 | 0 | 8 | 5 | 9 | 233 | 15 | 0 | 0 | 0 |
| Kenya | 1 | 1 | 4 | 2 | 10 | 0 | 3 | 2 | 1 | 7 | 4 | 0 | 0 | 1 |
| Kyrgyz Republic | 0 | 0 | 0 | 0 | 7 | 0 | 1 | 0 | 0 | 0 | 1 | 0 | 0 | 0 |
| Liberia | 1 | 0 | 0 | 0 | 3 | 0 | 0 | 1 | 0 | 2 | 1 | 0 | 0 | 1 |
| Myanmar | 3 | 0 | 17 | 2 | 456 | 1 | 2 | 1 | 2 | 64 | 6 | 14 | 0 | 3 |
| Nepal | 5 | 0 | 5 | 0 | 17 | 4 | 5 | 1 | 7 | 8 | 6 | 1 | 0 | 2 |
| Senegal | 2 | 0 | 2 | 2 | 1 | 0 | 8 | 0 | 0 | 10 | 22 | 0 | 0 | 1 |
| Somalia | 0 | 0 | 0 | 0 | 3 | 0 | 0 | 0 | 0 | 0 | 1 | 0 | 0 | 0 |
| Tajikistan | 0 | 0 | 1 | 0 | 8 | 1 | 1 | 0 | 0 | 2 | 0 | 0 | 0 | 0 |
| Tanzania | 0 | 0 | 0 | 0 | 1 | 0 | 1 | 0 | 1 | 1 | 2 | 0 | 0 | 0 |
| Uganda | 2 | 0 | 6 | 0 | 18 | 1 | 5 | 2 | 0 | 5 | 8 | 0 | 0 | 1 |
| Uzbekistan | 0 | 0 | 1 | 0 | 8 | 0 | 2 | 0 | 1 | 0 | 0 | 0 | 0 | 0 |
| Vietnam | 1 | 0 | 6 | 7 | 545 | 6 | 1 | 0 | 4 | 107 | 14 | 5 | 0 | 0 |
| Yemen | 0 | 0 | 0 | 0 | 0 | 0 | 0 | 0 | 0 | 0 | 0 | 0 | 0 | 1 |
| Zimbabwe | 4 | 1 | 4 | 2 | 7 | 1 | 2 | 2 | 2 | 8 | 3 | 1 | 1 | 1 |

| Country/State | Alabama | Alaska | Arizona | Arkansas | California | Colorado | Conn. | Delaware | District of Columbia | Florida | Georgia | Hawaii | Idaho | Iowa |
| --- | --- | --- | --- | --- | --- | --- | --- | --- | --- | --- | --- | --- | --- | --- |
| B. Lower Middle Income |  |  |  |  |  |  |  |  |  |  |  |  |  |  |
| Albania | 0 | 0 | 4 | 0 | 2 | 0 | 2 | 0 | 0 | 4 | 0 | 0 | 0 | 0 |
| Armenia | 0 | 0 | 2 | 0 | 195 | 1 | 3 | 0 | 0 | 3 | 1 | 0 | 0 | 0 |
| Belize | 0 | 0 | 8 | 3 | 6 | 1 | 1 | 1 | 0 | 27 | 12 | 1 | 0 | 1 |
| Bolivia | 6 | 0 | 8 | 1 | 29 | 0 | 2 | 2 | 2 | 55 | 6 | 0 | 1 | 1 |
| Bulgaria | 1 | 0 | 6 | 9 | 46 | 2 | 7 | 3 | 4 | 37 | 6 | 2 | 1 | 4 |
| Cameroon | 1 | 0 | 0 | 0 | 2 | 0 | 1 | 0 | 2 | 0 | 2 | 0 | 0 | 0 |
| China | 39 | 2 | 51 | 16 | 883 | 17 | 93 | 19 | 12 | 182 | 97 | 18 | 2 | 31 |
| Congo, Dem. Rep. of | 0 | 0 | 0 | 0 | 0 | 1 | 0 | 0 | 0 | 0 | 1 | 0 | 0 | 0 |
| Ecuador | 8 | 0 | 6 | 1 | 35 | 3 | 11 | 1 | 2 | 155 | 20 | 1 | 0 | 5 |
| Egypt | 39 | 0 | 50 | 25 | 768 | 11 | 45 | 20 | 16 | 446 | 81 | 6 | 1 | 40 |
| El Salvador | 2 | 0 | 5 | 1 | 60 | 0 | 3 | 0 | 2 | 59 | 10 | 0 | 0 | 1 |
| Georgia | 1 | 0 | 0 | 1 | 18 | 0 | 7 | 4 | 1 | 5 | 16 | 0 | 0 | 0 |
| Guatemala | 11 | 1 | 10 | 4 | 38 | 5 | 5 | 2 | 0 | 102 | 9 | 0 | 0 | 3 |
| Guyana | 3 | 0 | 0 | 0 | 0 | 0 | 0 | 0 | 1 | 1 | 7 | 0 | 0 | 0 |
| Honduras | 1 | 0 | 4 | 1 | 11 | 4 | 0 | 1 | 0 | 28 | 7 | 0 | 1 | 0 |
| India | 479 | 7 | 842 | 264 | 4869 | 173 | 815 | 171 | 184 | 2808 | 1246 | 48 | 14 | 370 |
| Indonesia | 2 | 0 | 5 | 1 | 68 | 0 | 3 | 0 | 0 | 20 | 3 | 5 | 0 | 0 |
| Iran | 17 | 0 | 59 | 3 | 1502 | 14 | 93 | 23 | 45 | 214 | 53 | 2 | 1 | 23 |
| Iraq | 9 | 0 | 14 | 2 | 160 | 2 | 12 | 1 | 4 | 40 | 9 | 0 | 1 | 10 |
| Jordan | 19 | 1 | 34 | 14 | 24 | 6 | 10 | 6 | 5 | 46 | 15 | 0 | 0 | 40 |
| Kazakhstan | 0 | 0 | 0 | 0 | 1 | 0 | 0 | 1 | 1 | 1 | 0 | 0 | 0 | 0 |
| Kosovo | 0 | 0 | 1 | 0 | 1 | 0 | 0 | 0 | 0 | 0 | 0 | 0 | 0 | 0 |
| Morocco | 1 | 0 | 2 | 0 | 14 | 0 | 4 | 0 | 3 | 7 | 0 | 0 | 0 | 1 |

| Country/State | Alabama | Alaska | Arizona | Arkansas | California | Colorado | Conn. | Delaware | District of Columbia | Florida | Georgia | Hawaii | Idaho | Iowa |
| --- | --- | --- | --- | --- | --- | --- | --- | --- | --- | --- | --- | --- | --- | --- |
| B. Lower Middle Income, cont’d |  |  |  |  |  |  |  |  |  |  |  |  |  |  |
| Nicaragua | 3 | 0 | 3 | 0 | 19 | 1 | 0 | 0 | 0 | 151 | 0 | 0 | 0 | 1 |
| Nigeria | 48 | 0 | 45 | 22 | 166 | 11 | 54 | 19 | 45 | 110 | 277 | 0 | 2 | 21 |
| Pakistan | 137 | 0 | 214 | 113 | 785 | 21 | 193 | 40 | 33 | 662 | 258 | 10 | 7 | 80 |
| Paraguay | 4 | 0 | 4 | 1 | 13 | 0 | 2 | 5 | 2 | 43 | 8 | 1 | 0 | 2 |
| Philippines | 147 | 10 | 329 | 77 | 3277 | 65 | 251 | 135 | 54 | 1407 | 292 | 176 | 6 | 83 |
| Sri Lanka | 0 | 0 | 11 | 3 | 192 | 0 | 46 | 5 | 2 | 42 | 17 | 1 | 0 | 4 |
| Sudan | 5 | 0 | 17 | 1 | 12 | 2 | 3 | 0 | 9 | 18 | 13 | 0 | 0 | 4 |
| Syria | 45 | 1 | 76 | 44 | 266 | 8 | 27 | 7 | 23 | 232 | 66 | 1 | 0 | 33 |
| Thailand | 19 | 0 | 19 | 4 | 254 | 8 | 12 | 4 | 6 | 83 | 20 | 20 | 0 | 23 |
| Tunisia | 0 | 0 | 1 | 0 | 3 | 0 | 0 | 0 | 0 | 3 | 0 | 0 | 0 | 0 |
| Ukraine | 9 | 0 | 13 | 7 | 219 | 5 | 19 | 1 | 4 | 72 | 14 | 7 | 0 | 12 |
|  |  |  |  |  |  |  |  |  |  |  |  |  |  |  |
| Total | 1113 | 24 | 1946 | 646 | 15201 | 382 | 1821 | 495 | 538 | 7622 | 2745 | 321 | 39 | 818 |
|  |  |  |  |  |  |  |  |  |  |  |  |  |  |  |
| IMG % of all state physicians | 11.0% | 1.6% | 14.3% | 11.1% | 15.2% | 3.0% | 13.8% | 22.8% | 11.2% | 16.7% | 13.1% | 8.0% | 1.5% | 14.5% |

| Country/State | Illinois | Indiana | Kansas | Kentucky | Louisiana | Maine | Maryland | Mass. | Michigan | Minn. | Miss’ippi | Missouri | Montana | Nebraska |
| --- | --- | --- | --- | --- | --- | --- | --- | --- | --- | --- | --- | --- | --- | --- |
| A. Lower Income |  |  |  |  |  |  |  |  |  |  |  |  |  |  |
| Afghanistan | 2 | 8 | 2 | 2 | 0 | 0 | 4 | 6 | 2 | 1 | 0 | 4 | 0 | 2 |
| Bangladesh | 45 | 9 | 15 | 20 | 23 | 1 | 43 | 33 | 65 | 14 | 15 | 24 | 0 | 5 |
| Ethiopia | 20 | 5 | 8 | 3 | 7 | 1 | 95 | 5 | 19 | 15 | 1 | 12 | 0 | 1 |
| Ghana | 43 | 10 | 5 | 6 | 13 | 1 | 50 | 12 | 26 | 9 | 4 | 10 | 0 | 2 |
| Haiti | 46 | 4 | 2 | 1 | 13 | 1 | 30 | 23 | 11 | 0 | 1 | 18 | 0 | 2 |
| Kenya | 11 | 10 | 4 | 2 | 4 | 0 | 2 | 5 | 3 | 9 | 0 | 11 | 0 | 1 |
| Kyrgyz Republic | 0 | 1 | 0 | 2 | 0 | 0 | 1 | 1 | 2 | 1 | 0 | 1 | 0 | 0 |
| Liberia | 1 | 7 | 0 | 0 | 2 | 0 | 7 | 1 | 2 | 0 | 1 | 0 | 0 | 0 |
| Myanmar | 50 | 5 | 3 | 6 | 2 | 0 | 65 | 13 | 8 | 4 | 0 | 6 | 0 | 3 |
| Nepal | 57 | 3 | 3 | 6 | 8 | 3 | 11 | 21 | 24 | 11 | 0 | 9 | 0 | 5 |
| Senegal | 19 | 1 | 0 | 3 | 10 | 2 | 6 | 0 | 9 | 0 | 1 | 2 | 0 | 0 |
| Somalia | 0 | 0 | 0 | 0 | 0 | 0 | 0 | 0 | 1 | 5 | 0 | 0 | 0 | 0 |
| Tajikistan | 0 | 1 | 0 | 0 | 0 | 0 | 1 | 2 | 1 | 0 | 0 | 1 | 0 | 0 |
| Tanzania | 1 | 1 | 0 | 0 | 1 | 0 | 2 | 1 | 1 | 5 | 0 | 0 | 0 | 0 |
| Uganda | 9 | 2 | 2 | 1 | 4 | 0 | 10 | 5 | 9 | 7 | 1 | 1 | 0 | 1 |
| Uzbekistan | 2 | 3 | 0 | 1 | 0 | 0 | 2 | 8 | 4 | 1 | 0 | 1 | 0 | 0 |
| Vietnam | 15 | 3 | 4 | 8 | 27 | 1 | 15 | 26 | 12 | 6 | 3 | 7 | 0 | 5 |
| Yemen | 0 | 0 | 0 | 1 | 0 | 0 | 0 | 0 | 5 | 0 | 0 | 0 | 0 | 0 |
| Zimbabwe | 4 | 3 | 0 | 2 | 0 | 0 | 8 | 4 | 5 | 0 | 2 | 1 | 0 | 0 |

| Country/State | Illinois | Indiana | Kansas | Kentucky | Louisiana | Maine | Maryland | Mass. | Michigan | Minn. | Miss’ippi | Missouri | Montana | Nebraska |
| --- | --- | --- | --- | --- | --- | --- | --- | --- | --- | --- | --- | --- | --- | --- |
| B. Lower Middle Income |  |  |  |  |  |  |  |  |  |  |  |  |  |  |
| Albania | 9 | 0 | 0 | 0 | 0 | 0 | 2 | 9 | 4 | 1 | 0 | 1 | 0 | 0 |
| Armenia | 9 | 1 | 1 | 2 | 1 | 1 | 3 | 9 | 5 | 1 | 0 | 2 | 0 | 0 |
| Belize | 9 | 1 | 0 | 0 | 8 | 0 | 0 | 1 | 8 | 2 | 2 | 1 | 0 | 0 |
| Bolivia | 73 | 6 | 4 | 5 | 4 | 0 | 10 | 10 | 4 | 1 | 0 | 3 | 0 | 0 |
| Bulgaria | 55 | 12 | 2 | 9 | 6 | 4 | 18 | 22 | 22 | 15 | 2 | 7 | 0 | 2 |
| Cameroon | 2 | 0 | 1 | 0 | 0 | 0 | 7 | 5 | 8 | 0 | 0 | 0 | 0 | 0 |
| China | 199 | 89 | 29 | 32 | 58 | 5 | 235 | 251 | 152 | 59 | 12 | 117 | 0 | 26 |
| Congo, Dem. Rep. of | 1 | 0 | 0 | 0 | 0 | 0 | 1 | 0 | 0 | 0 | 0 | 3 | 0 | 0 |
| Ecuador | 31 | 6 | 4 | 9 | 12 | 0 | 11 | 13 | 12 | 9 | 2 | 11 | 0 | 3 |
| Egypt | 231 | 68 | 36 | 57 | 55 | 8 | 149 | 187 | 251 | 43 | 22 | 70 | 2 | 16 |
| El Salvador | 15 | 1 | 0 | 2 | 7 | 0 | 8 | 2 | 6 | 0 | 2 | 2 | 0 | 1 |
| Georgia | 8 | 1 | 3 | 2 | 2 | 0 | 6 | 4 | 4 | 1 | 3 | 4 | 0 | 0 |
| Guatemala | 29 | 4 | 2 | 3 | 9 | 2 | 9 | 24 | 13 | 10 | 2 | 11 | 0 | 0 |
| Guyana | 2 | 0 | 0 | 0 | 1 | 0 | 0 | 0 | 1 | 0 | 0 | 1 | 0 | 0 |
| Honduras | 9 | 6 | 0 | 2 | 6 | 0 | 5 | 4 | 2 | 1 | 1 | 3 | 1 | 0 |
| India | 3889 | 941 | 392 | 660 | 659 | 99 | 1562 | 1542 | 2916 | 592 | 218 | 1067 | 8 | 271 |
| Indonesia | 11 | 2 | 2 | 0 | 4 | 0 | 5 | 1 | 8 | 2 | 2 | 2 | 0 | 0 |
| Iran | 236 | 49 | 23 | 38 | 42 | 12 | 272 | 140 | 151 | 41 | 6 | 63 | 1 | 7 |
| Iraq | 111 | 22 | 10 | 10 | 10 | 4 | 52 | 23 | 259 | 7 | 0 | 19 | 0 | 4 |
| Jordan | 70 | 31 | 12 | 19 | 7 | 2 | 11 | 23 | 111 | 13 | 1 | 49 | 0 | 15 |
| Kazakhstan | 0 | 0 | 0 | 0 | 0 | 0 | 0 | 0 | 0 | 0 | 0 | 0 | 0 | 0 |
| Kosovo | 5 | 0 | 0 | 0 | 0 | 0 | 0 | 1 | 0 | 0 | 0 | 0 | 0 | 0 |
| Morocco | 4 | 1 | 1 | 0 | 2 | 0 | 2 | 6 | 3 | 3 | 0 | 0 | 0 | 0 |

| Country/State | Illinois | Indiana | Kansas | Kentucky | Louisiana | Maine | Maryland | Mass. | Michigan | Minn. | Miss’ippi | Missouri | Montana | Nebraska |
| --- | --- | --- | --- | --- | --- | --- | --- | --- | --- | --- | --- | --- | --- | --- |
| B. Lower Middle Income, cont’d |  |  |  |  |  |  |  |  |  |  |  |  |  |  |
| Nicaragua | 16 | 3 | 1 | 0 | 7 | 0 | 8 | 3 | 2 | 3 | 4 | 2 | 1 | 0 |
| Nigeria | 193 | 67 | 16 | 34 | 56 | 4 | 198 | 41 | 94 | 62 | 40 | 54 | 0 | 13 |
| Pakistan | 860 | 238 | 112 | 243 | 146 | 21 | 385 | 273 | 687 | 189 | 75 | 359 | 1 | 51 |
| Paraguay | 6 | 2 | 2 | 2 | 3 | 0 | 5 | 10 | 21 | 4 | 2 | 5 | 0 | 0 |
| Philippines | 1417 | 390 | 147 | 252 | 119 | 40 | 640 | 282 | 749 | 142 | 62 | 337 | 8 | 23 |
| Sri Lanka | 31 | 8 | 6 | 9 | 4 | 3 | 34 | 19 | 32 | 7 | 0 | 13 | 0 | 2 |
| Sudan | 18 | 9 | 3 | 1 | 1 | 1 | 13 | 8 | 48 | 14 | 4 | 10 | 0 | 0 |
| Syria | 336 | 129 | 46 | 93 | 58 | 10 | 56 | 90 | 511 | 29 | 16 | 96 | 2 | 13 |
| Thailand | 209 | 47 | 12 | 17 | 14 | 3 | 81 | 36 | 78 | 28 | 11 | 66 | 0 | 7 |
| Tunisia | 3 | 0 | 0 | 0 | 2 | 0 | 1 | 5 | 4 | 0 | 0 | 0 | 0 | 0 |
| Ukraine | 91 | 12 | 2 | 9 | 11 | 4 | 37 | 71 | 41 | 16 | 2 | 19 | 0 | 0 |
|  |  |  |  |  |  |  |  |  |  |  |  |  |  |  |
| Total | 8513 | 2222 | 917 | 1574 | 1428 | 233 | 4178 | 3281 | 6416 | 1383 | 520 | 2505 | 24 | 481 |
|  |  |  |  |  |  |  |  |  |  |  |  |  |  |  |
| IMG % of all state physicians | 23.7% | 16.1% | 14.6% | 16.0% | 12.2% | 6.5% | 17.7% | 10.7% | 25.5% | 8.7% | 10.0% | 17.3% | 1.1% | 10.9% |

| Country/State | Nevada | New Hamp. | New Jersey | New Mexico | New York | North Carolina | North Dakota | Ohio | Okla. | Oregon | Penn. | Rhode Island | South Carolina | South Dakota |
| --- | --- | --- | --- | --- | --- | --- | --- | --- | --- | --- | --- | --- | --- | --- |
| A. Lower Income |  |  |  |  |  |  |  |  |  |  |  |  |  |  |
| Afghanistan | 0 | 0 | 8 | 1 | 17 | 3 | 0 | 4 | 1 | 1 | 5 | 0 | 1 | 0 |
| Bangladesh | 9 | 8 | 60 | 1 | 417 | 47 | 3 | 55 | 7 | 10 | 71 | 4 | 7 | 0 |
| Ethiopia | 0 | 0 | 11 | 0 | 59 | 14 | 1 | 14 | 4 | 0 | 17 | 0 | 2 | 0 |
| Ghana | 22 | 2 | 38 | 4 | 101 | 26 | 1 | 46 | 2 | 1 | 21 | 4 | 16 | 1 |
| Haiti | 0 | 3 | 56 | 1 | 500 | 9 | 0 | 8 | 1 | 1 | 16 | 5 | 2 | 0 |
| Kenya | 1 | 1 | 8 | 0 | 11 | 6 | 1 | 6 | 4 | 1 | 4 | 2 | 3 | 3 |
| Kyrgyz Republic | 0 | 0 | 3 | 0 | 5 | 1 | 0 | 0 | 0 | 0 | 1 | 0 | 0 | 0 |
| Liberia | 0 | 0 | 3 | 1 | 3 | 3 | 1 | 3 | 1 | 0 | 2 | 0 | 1 | 0 |
| Myanmar | 9 | 5 | 36 | 3 | 235 | 7 | 6 | 14 | 6 | 5 | 41 | 0 | 2 | 0 |
| Nepal | 0 | 4 | 7 | 2 | 123 | 8 | 4 | 33 | 5 | 4 | 54 | 4 | 4 | 4 |
| Senegal | 2 | 1 | 4 | 1 | 15 | 3 | 0 | 9 | 1 | 0 | 7 | 4 | 5 | 0 |
| Somalia | 0 | 0 | 0 | 0 | 2 | 0 | 0 | 1 | 0 | 0 | 0 | 0 | 0 | 0 |
| Tajikistan | 0 | 0 | 1 | 0 | 32 | 0 | 0 | 0 | 0 | 1 | 2 | 0 | 0 | 0 |
| Tanzania | 2 | 0 | 1 | 1 | 1 | 0 | 0 | 1 | 1 | 0 | 0 | 0 | 0 | 0 |
| Uganda | 2 | 2 | 7 | 0 | 18 | 7 | 2 | 12 | 2 | 1 | 16 | 0 | 3 | 0 |
| Uzbekistan | 0 | 0 | 6 | 0 | 75 | 0 | 0 | 4 | 0 | 0 | 3 | 0 | 0 | 0 |
| Vietnam | 2 | 0 | 13 | 3 | 20 | 8 | 1 | 7 | 13 | 6 | 27 | 0 | 1 | 0 |
| Yemen | 0 | 0 | 2 | 0 | 4 | 0 | 0 | 1 | 0 | 0 | 0 | 0 | 0 | 0 |
| Zimbabwe | 2 | 0 | 2 | 1 | 8 | 3 | 4 | 5 | 2 | 0 | 6 | 0 | 2 | 2 |

| Country/State | Nevada | New Hamp. | New Jersey | New Mexico | New York | North Carolina | North Dakota | Ohio | Okla. | Oregon | Penn. | Rhode Island | South Carolina | South Dakota |
| --- | --- | --- | --- | --- | --- | --- | --- | --- | --- | --- | --- | --- | --- | --- |
| B. Lower Middle Income |  |  |  |  |  |  |  |  |  |  |  |  |  |  |
| Albania | 0 | 1 | 1 | 0 | 33 | 0 | 0 | 8 | 1 | 0 | 2 | 0 | 2 | 0 |
| Armenia | 1 | 0 | 5 | 0 | 30 | 3 | 2 | 6 | 1 | 0 | 10 | 0 | 2 | 1 |
| Belize | 4 | 0 | 7 | 1 | 16 | 4 | 0 | 14 | 8 | 0 | 3 | 1 | 1 | 1 |
| Bolivia | 0 | 0 | 13 | 6 | 23 | 3 | 0 | 17 | 2 | 3 | 11 | 2 | 2 | 0 |
| Bulgaria | 6 | 2 | 24 | 4 | 97 | 10 | 3 | 18 | 2 | 6 | 26 | 2 | 5 | 2 |
| Cameroon | 1 | 1 | 2 | 2 | 5 | 2 | 0 | 1 | 0 | 0 | 3 | 0 | 0 | 0 |
| China | 22 | 16 | 359 | 13 | 912 | 84 | 8 | 223 | 38 | 51 | 294 | 19 | 27 | 5 |
| Congo, Dem. Rep. of | 0 | 1 | 0 | 0 | 3 | 1 | 0 | 0 | 1 | 0 | 0 | 0 | 1 | 0 |
| Ecuador | 2 | 2 | 31 | 3 | 96 | 6 | 0 | 21 | 9 | 0 | 21 | 5 | 3 | 2 |
| Egypt | 16 | 13 | 422 | 11 | 673 | 84 | 10 | 385 | 48 | 19 | 216 | 34 | 40 | 10 |
| El Salvador | 3 | 0 | 11 | 3 | 34 | 2 | 0 | 6 | 0 | 0 | 9 | 7 | 2 | 0 |
| Georgia | 1 | 1 | 9 | 0 | 65 | 0 | 1 | 15 | 1 | 0 | 4 | 0 | 2 | 0 |
| Guatemala | 1 | 0 | 6 | 10 | 36 | 11 | 0 | 10 | 1 | 1 | 18 | 1 | 3 | 0 |
| Guyana | 0 | 0 | 0 | 0 | 8 | 1 | 0 | 0 | 1 | 0 | 1 | 0 | 0 | 0 |
| Honduras | 1 | 0 | 5 | 0 | 11 | 3 | 0 | 2 | 4 | 1 | 3 | 1 | 0 | 0 |
| India | 278 | 124 | 3065 | 166 | 6631 | 956 | 151 | 2798 | 384 | 203 | 3173 | 139 | 326 | 50 |
| Indonesia | 4 | 1 | 18 | 1 | 29 | 4 | 1 | 9 | 2 | 0 | 19 | 1 | 0 | 0 |
| Iran | 35 | 7 | 199 | 12 | 521 | 38 | 4 | 183 | 15 | 11 | 267 | 21 | 11 | 1 |
| Iraq | 2 | 3 | 46 | 5 | 100 | 9 | 0 | 49 | 4 | 6 | 51 | 6 | 3 | 1 |
| Jordan | 3 | 1 | 58 | 12 | 59 | 14 | 3 | 83 | 16 | 1 | 29 | 20 | 8 | 3 |
| Kazakhstan | 0 | 1 | 1 | 0 | 7 | 0 | 0 | 0 | 0 | 0 | 3 | 0 | 0 | 0 |
| Kosovo | 0 | 0 | 3 | 0 | 6 | 1 | 0 | 4 | 0 | 0 | 0 | 0 | 1 | 0 |
| Morocco | 1 | 0 | 2 | 0 | 15 | 2 | 0 | 4 | 0 | 0 | 5 | 0 | 0 | 0 |

| Country/State | Nevada | New Hamp. | New Jersey | New Mexico | New York | North Carolina | North Dakota | Ohio | Okla. | Oregon | Penn. | Rhode Island | South Carolina | South Dakota |
| --- | --- | --- | --- | --- | --- | --- | --- | --- | --- | --- | --- | --- | --- | --- |
| B. Lower Middle Income, cont’d |  |  |  |  |  |  |  |  |  |  |  |  |  |  |
| Nicaragua | 0 | 0 | 13 | 3 | 11 | 0 | 0 | 3 | 3 | 1 | 0 | 0 | 0 | 0 |
| Nigeria | 28 | 2 | 184 | 14 | 404 | 149 | 19 | 154 | 22 | 5 | 136 | 2 | 45 | 3 |
| Pakistan | 95 | 24 | 558 | 51 | 1469 | 181 | 35 | 569 | 143 | 31 | 594 | 79 | 67 | 25 |
| Paraguay | 1 | 0 | 9 | 2 | 37 | 2 | 0 | 3 | 2 | 1 | 8 | 0 | 4 | 0 |
| Philippines | 465 | 56 | 1483 | 59 | 2333 | 180 | 54 | 919 | 118 | 77 | 878 | 87 | 122 | 40 |
| Sri Lanka | 10 | 1 | 73 | 2 | 114 | 12 | 2 | 33 | 2 | 2 | 46 | 1 | 4 | 0 |
| Sudan | 0 | 2 | 9 | 0 | 14 | 10 | 0 | 22 | 1 | 2 | 13 | 0 | 1 | 0 |
| Syria | 14 | 7 | 140 | 13 | 234 | 35 | 8 | 384 | 38 | 14 | 187 | 16 | 19 | 6 |
| Thailand | 27 | 3 | 38 | 3 | 109 | 16 | 8 | 65 | 5 | 7 | 80 | 4 | 17 | 2 |
| Tunisia | 1 | 0 | 1 | 0 | 5 | 0 | 0 | 3 | 0 | 0 | 2 | 0 | 2 | 0 |
| Ukraine | 9 | 4 | 153 | 0 | 386 | 18 | 1 | 49 | 7 | 2 | 99 | 9 | 3 | 3 |
|  |  |  |  |  |  |  |  |  |  |  |  |  |  |  |
| Total | 1082 | 299 | 7214 | 415 | 16172 | 1986 | 334 | 6283 | 929 | 475 | 6504 | 480 | 772 | 165 |
|  |  |  |  |  |  |  |  |  |  |  |  |  |  |  |
| IMG % of all state physicians | 22.3% | 8.1% | 26.5% | 8.6% | 21.3% | 8.4% | 21.2% | 20.5% | 14.7% | 4.5% | 17.1% | 12.2% | 7.6% | 9.3% |

| Country/State | Tenn. | Texas | Utah | Vermont | Virginia | Wash. | West Virginia | Wisc. | Wyoming | TOTAL | IMGs/  million pop. |
| --- | --- | --- | --- | --- | --- | --- | --- | --- | --- | --- | --- |
| A. Lower Income |  |  |  |  |  |  |  |  |  |  |  |
| Afghanistan | 2 | 8 | 0 | 1 | 22 | 1 | 1 | 0 | 0 | 194 |  |
| Bangladesh | 38 | 86 | 2 | 0 | 76 | 7 | 12 | 10 | 1 | 1515 | 9·5 |
| Ethiopia | 9 | 20 | 1 | 0 | 63 | 7 | 12 | 8 | 0 | 542 | 6·7 |
| Ghana | 13 | 41 | 3 | 0 | 25 | 4 | 0 | 9 | 0 | 727 | 31·6 |
| Haiti | 7 | 7 | 0 | 0 | 6 | 1 | 2 | 1 | 0 | 1068 | 106·8 |
| Kenya | 2 | 15 | 0 | 0 | 3 | 8 | 0 | 2 | 0 | 179 | 4·6 |
| Kyrgyz Republic | 0 | 0 | 0 | 0 | 1 | 1 | 0 | 0 | 0 | 30 | 6·0 |
| Liberia | 1 | 2 | 0 | 0 | 3 | 0 | 0 | 2 | 0 | 56 | 14·0 |
| Myanmar | 5 | 55 | 1 | 0 | 20 | 7 | 3 | 5 | 0 | 1201 | 4·1 |
| Nepal | 6 | 11 | 1 | 0 | 17 | 3 | 0 | 9 | 0 | 525 | 18·1 |
| Senegal | 2 | 7 | 1 | 1 | 3 | 1 | 1 | 1 | 2 | 172 | 14·3 |
| Somalia | 0 | 0 | 0 | 0 | 1 | 0 | 0 | 0 | 0 | 14 | 1·6 |
| Tajikistan | 0 | 0 | 0 | 0 | 0 | 0 | 0 | 0 | 0 | 55 | 7·9 |
| Tanzania | 2 | 5 | 0 | 0 | 1 | 0 | 0 | 0 | 0 | 33 | 0·8 |
| Uganda | 4 | 10 | 0 | 0 | 8 | 5 | 2 | 9 | 0 | 210 | 6·6 |
| Uzbekistan | 0 | 0 | 0 | 0 | 2 | 0 | 0 | 0 | 0 | 124 | 4·6 |
| Vietnam | 6 | 135 | 1 | 0 | 58 | 28 | 2 | 4 | 0 | 1164 | 13·5 |
| Yemen | 0 | 0 | 0 | 0 | 1 | 0 | 0 | 0 | 0 | 15 | 0·7 |
| Zimbabwe | 2 | 10 | 0 | 0 | 1 | 3 | 0 | 1 | 0 | 122 | 10·2 |

| Country/State | Tenn. | Texas | Utah | Vermont | Virginia | Wash. | West Virginia | Wisc. | Wyoming | TOTAL | IMGs/  million pop. |
| --- | --- | --- | --- | --- | --- | --- | --- | --- | --- | --- | --- |
| B. Lower Middle Income |  |  |  |  |  |  |  |  |  |  |  |
| Albania | 1 | 3 | 0 | 0 | 2 | 0 | 0 | 0 | 0 | 92 | 30·7 |
| Armenia | 5 | 6 | 1 | 0 | 0 | 2 | 0 | 0 | 0 | 315 | 105·0 |
| Belize | 0 | 13 | 0 | 0 | 5 | 5 | 0 | 0 | 3 | 180 |  |
| Bolivia | 0 | 31 | 0 | 1 | 8 | 1 | 2 | 2 | 0 | 369 | 36·9 |
| Bulgaria | 18 | 28 | 1 | 0 | 9 | 9 | 0 | 14 | 2 | 592 | 74·0 |
| Cameroon | 3 | 7 | 0 | 0 | 4 | 0 | 0 | 1 | 0 | 63 | 3·3 |
| China | 81 | 348 | 9 | 6 | 111 | 139 | 15 | 78 | 0 | 5584 | 4·2 |
| Congo, Dem. Rep. of | 0 | 0 | 0 | 0 | 0 | 0 | 0 | 0 | 0 | 14 | 0·2 |
| Ecuador | 11 | 66 | 1 | 0 | 10 | 3 | 0 | 6 | 0 | 679 | 52·2 |
| Egypt | 71 | 272 | 4 | 3 | 155 | 49 | 52 | 55 | 3 | 5389 | 65·7 |
| El Salvador | 5 | 88 | 0 | 0 | 5 | 2 | 1 | 0 | 1 | 373 | 62·2 |
| Georgia | 1 | 3 | 0 | 2 | 7 | 3 | 0 | 2 | 0 | 208 | 52·0 |
| Guatemala | 9 | 113 | 2 | 0 | 9 | 7 | 2 | 5 | 0 | 558 | 39·9 |
| Guyana | 3 | 0 | 0 | 0 | 0 | 0 | 0 | 0 | 0 | 31 |  |
| Honduras | 3 | 20 | 1 | 0 | 4 | 1 | 0 | 4 | 1 | 166 | 23·7 |
| India | 791 | 3572 | 116 | 22 | 1177 | 403 | 418 | 793 | 13 | 52874 | 46·3 |
| Indonesia | 2 | 26 | 0 | 0 | 6 | 6 | 1 | 2 | 0 | 278 | 1·2 |
| Iran | 53 | 198 | 9 | 2 | 179 | 48 | 33 | 53 | 3 | 5034 | 69·9 |
| Iraq | 14 | 40 | 2 | 0 | 55 | 14 | 9 | 16 | 2 | 1232 |  |
| Jordan | 30 | 76 | 5 | 1 | 14 | 5 | 28 | 20 | 2 | 1075 | 179·2 |
| Kazakstan | 0 | 0 | 0 | 0 | 0 | 0 | 0 | 0 | 0 | 16 | 1·0 |
| Kosovo | 0 | 0 | 0 | 0 | 0 | 0 | 0 | 0 | 0 | 23 |  |
| Morocco | 1 | 4 | 0 | 0 | 4 | 0 | 0 | 1 | 0 | 93 | 3·0 |

| Country/State | Tenn. | Texas | Utah | Vermont | Virginia | Wash. | West Virginia | Wisc. | Wyoming | TOTAL | IMGs/  million pop. |
| --- | --- | --- | --- | --- | --- | --- | --- | --- | --- | --- | --- |
| Nicaragua | 2 | 46 | 1 | 0 | 6 | 1 | 0 | 5 | 0 | 330 | 55·0 |
| Nigeria | 109 | 300 | 3 | 1 | 71 | 19 | 12 | 38 | 1 | 3415 | 22·6 |
| Pakistan | 259 | 1196 | 17 | 12 | 364 | 81 | 169 | 213 | 5 | 12433 | 74·9 |
| Paraguay | 4 | 30 | 0 | 0 | 6 | 2 | 3 | 1 | 0 | 263 | 4·4 |
| Philippines | 209 | 902 | 19 | 6 | 603 | 320 | 316 | 362 | 9 | 20625 | 229·2 |
| Sri Lanka | 7 | 29 | 0 | 1 | 23 | 12 | 7 | 7 | 0 | 880 | 44·0 |
| Sudan | 6 | 14 | 0 | 0 | 25 | 1 | 2 | 17 | 0 | 353 | 8·6 |
| Syria | 62 | 203 | 13 | 2 | 66 | 24 | 80 | 71 | 3 | 3953 | 188·2 |
| Thailand | 20 | 103 | 0 | 0 | 28 | 28 | 20 | 22 | 0 | 1688 | 25·2 |
| Tunisia | 0 | 3 | 0 | 0 | 5 | 0 | 1 | 0 | 0 | 45 | 4·5 |
| Ukraine | 8 | 34 | 2 | 2 | 29 | 22 | 2 | 19 | 0 | 1560 | 33·9 |
|  |  |  |  |  |  |  |  |  |  |  |  |
| Total | 1887 | 8186 | 216 | 63 | 3301 | 1283 | 1208 | 1868 | 51 | 128729 |  |
|  |  |  |  |  |  |  |  |  |  |  |  |
| IMG % of all state physicians | 11.5% | 15.7% | 3.8% | 2.7% | 15.5% | 7.2% | 28.7% | 12.8% | 5.1% | 15.7% |  |
